# Epigenetic silencing of the *NR4A3* tumor suppressor, by aberrant JAK/STAT signaling, predicts prognosis in gastric cancer

**DOI:** 10.1038/srep31690

**Published:** 2016-08-16

**Authors:** Chung-Min Yeh, Liang-Yu Chang, Shu-Hui Lin, Jian-Liang Chou, Hsiao-Yen Hsieh, Li-Han Zeng, Sheng-Yu Chuang, Hsiao-Wen Wang, Claudia Dittner, Cheng-Yu Lin, Jora M. J. Lin, Yao-Ting Huang, Enders K. W. Ng, Alfred S. L. Cheng, Shu-Fen Wu, Jiayuh Lin, Kun-Tu Yeh, Michael W. Y. Chan

**Affiliations:** 1 Department of Surgical Pathology, Changhua Christian Hospital, Changhua, Taiwan; 2 Department of Medical Technology, Jen-Teh Junior College of Medicine, Nursing and Management, Miaoli, Taiwan; 3 Department of Life Science, National Chung Cheng University, Min-Hsiung, Chia-Yi, Taiwan; 4 Institute of Molecular Biology, National Chung Cheng University, Min-Hsiung, Chia-Yi, Taiwan; 5 Institute of Medicine, Chung Shan Medical University, Taichung, Taiwan; 6 Department of Medical Research, Ditmanson Medical Foundation Chiayi Christian Hospital, Chia-Yi, Taiwan; 7 Department of Computer Science, National Chung Cheng University, Min-Hsiung, Chia-Yi, Taiwan; 8 Zentrum für Molekulare Biologie Heidelberg (ZMBH), DKFZ-ZMBH Alliance, Heidelberg, Germany; 9 Department of Surgery, The Chinese University of Hong Kong, Hong Kong SAR, China; 10 School of Biomedical Sciences, The Chinese University of Hong Kong, Hong Kong SAR, China; 11 Department of Pediatrics, The Ohio State University, Columbus, OH, USA; 12 School of Medicine, Chung Shan Medical University, Taichung, Taiwan

## Abstract

While aberrant JAK/STAT signaling is crucial to the development of gastric cancer (GC), its effects on epigenetic alterations of its transcriptional targets remains unclear. In this study, by expression microarrays coupled with bioinformatic analyses, we identified a putative STAT3 target gene, *NR4A3* that was downregulated in MKN28 GC daughter cells overexpressing a constitutively activated STAT3 mutant (S16), as compared to an empty vector control (C9). Bisulphite pyrosequencing and demethylation treatment showed that *NR4A3* was epigenetically silenced by promoter DNA methylation in S16 and other GC cell lines including AGS cells, showing constitutive activation of STAT3. Subsequent experiments revealed that *NR4A3* promoter binding by STAT3 might repress its transcription. Long-term depletion of STAT3 derepressed *NR4A3* expression, by promoter demethylation, in AGS GC cells. *NR4A3* re-expression in GC cell lines sensitized the cells to cisplatin, and inhibited tumor growth *in vitro* and *in vivo*, in an animal model. Clinically, GC patients with high *NR4A3* methylation, or lower NR4A3 protein expression, had significantly shorter overall survival. Intriguingly, STAT3 activation significantly associated only with *NR4A3* methylation in low-stage patient samples. Taken together, aberrant JAK/STAT3 signaling epigenetically silences a potential tumor suppressor, *NR4A3,* in gastric cancer, plausibly representing a reliable biomarker for gastric cancer prognosis.

Gastric cancer (GC) is the third leading cause of cancer death worldwide^1^. About 90% of GCs are adenocarcinomas, which can be classified into poorly differentiated diffuse, well-differentiated intestinal, and mixed types^2^. Due to the lack effective therapy, the prognosis of patients with GC remains poor with a 5-year overall survival of less than 25%^3^. Infection by *Helicobacter pylori (H. pylori*), a gram-negative bacillus, is considered to be the strongest risk factor for gastric carcinogenesis^4^. Patients infected with c*ytotoxin-associated gene A (CagA*)-positive *H. pylori* have a much higher risk for atrophic gastritis, as well as gastric cancer^5^,^6^,^7^. Once the stomach epithelial cells are infected by *H. pylori*, the CagA protein is injected into the host cells, resulting in multiple signaling pathway dysregulation, including activation of JAK/STAT signaling^8^,^9^,^10^,^11^. Moreover, infection of *H. pylori* associates with increased cytokine expression in particularly, interleukin-6, (IL-6), and robust inflammatory response, in gastric cancer^12^,^13^, thus suggesting that activation of IL-6-JAK/STAT3 signaling pathways may be crucial for GC development.

JAK/STAT signaling is involved in host defense as well as cancer development^14^,^15^,^16^. Several studies now indicate that STAT3 activation is crucial for GC initiation and progression^17^,^18^. Upon binding of IL-6 to its transmembrane receptor, the cytoplasmic tyrosine kinase, Janus kinase (JAK), is activated, followed by phosphorylation and dimerization of STAT3^19^. P-STAT3 then translocates to the nucleus and binds to specific DNA sequence to regulate transcription of specific target genes. Other studies have also demonstrated that STAT3 activation is more prominent in GC patients infected with CagA-positive *H. pylori*^20^, although the exact role of JAK/STAT signaling in GC is not fully understood.

Epigenetic modifications including DNA methylation, are essential gene-regulatory event^21^. In the human genome, methylation takes place at the 5′ position of cytosine in CG dinucleotides resulting in the formation of 5-methylcytosine (5mC) which is initiated and maintained by DNA methyltransferases (DNMTs). Between 60 to 90% of the cytosine at CG dinucleotides are methylated under physiological conditions. In contrast, cytosines that are found in a cluster of CpG rich regions, or “CpG islands”, within the promoter regions of tumor suppressor genes, are typically protected from methylation in normal somatic cells^22^. However, numerous studies including our own, have demonstrated that tumor suppressor genes are frequently silenced by promoter hypermethylation in human cancers, including GC^23^,^24^,^25^,^26^,^27^,^28^, leading to this event being considered a “hallmark of cancer”^21^. However, the mechanism of how aberrant promoter methylation arises is not fully understood.

Relatedly, we recently found that dysregulation of TGF-β signaling may lead to epigenetic silencing of its targets in ovarian cancer^22^,^29^. To examine the existence of such “epigenetic-mediated transcriptional silencing” in GC, we investigated how STAT3 activation influences the epigenetic silencing of STAT3 targets. To that end, we performed gene expression microarray data, together with bioinformatic analysis, to identify STAT3 targets that are epigenetically silenced by promoter methylation in GC. Our results showed that a nuclear receptor, *NR4A3,* was epigenetically silenced by promoter DNA methylation in GC cells with constitutive STAT3 activation. The clinical significance of P-STAT3-mediated methylation of *NR4A3*, in GC patients, was also investigated.

## Results

### Generation and characterization of a constitutively activated STAT3 gastric cancer cell line

**Figure 1:**
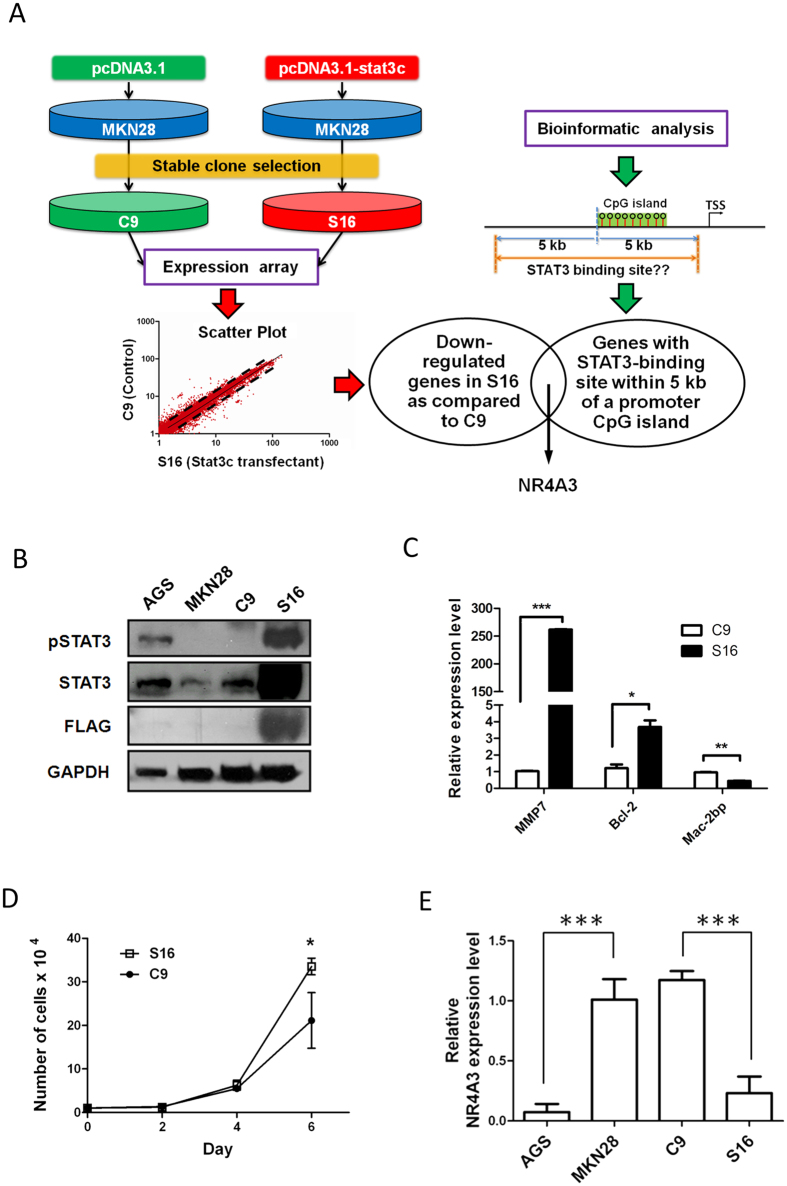
Integrated expression microarray and bioinformatic analyses identifies *NR4A3* as an epigenetically silenced target of STAT3 in gastric cancer. (**A**) Schematic diagram showing the experimental scheme of this study. Empty vector (pcDNA 3.1) or vector expressing a constitutively activated mouse *Stat3* mutant (pcDNA3.1-stat3c) were transfected into MKN28 gastric cancer (GC) cells. Stable transfectants were selected from cells expressing the empty vector control (C9) or the *Stat3* mutant (S16). Total RNA from C9 and S16 cells was then extracted for expression microarray analysis using an Agilent Human Whole Genome 44 K Expression Microarray, with data presented as a scatter plot. Each dot of the scatter plot represents the fluorescence signal (i.e., the mRNA expression level) of each gene on the array in C9 vs. S16 cells. Genes outside of the black dotted lines denote expression changes of ≥1.5-fold. In addition, bioinformatic analysis was performed to identify potential STAT3 targets by filtering genes with at least one STAT3-binding site within 5 kb of the promoter CpG island, revealing 526 putative STAT3 target genes. One such downregulated STAT3 target in S16 cells, *NR4A3*, was selected for further analysis. (**B**) Western blot was performed to investigate the activation status of STAT3 in C9, S16, and the parental MKN28 GC cells. AGS GC cells showing constitutive STAT3 activation were used as a positive control. *Stat3c* stable transfectants (S16) were confirmed by hybridization of an anti-FLAG antibody. (**C**) STAT3 activation in S16 cells was confirmed by the expression of STAT3 targets *MMP7, Bcl-2* and *Mac-2bp*, using qRT-PCR (***P < 0.001; **P < 0.01; *P < 0.05). Both S16 and AGS cells showed *MMP7* upregulation, compared to C9 control and MKN28 GC cells. (**D**) *STAT3C* overexpression slightly increased cell growth in MKN28 cells. S16 and C9 cell numbers were measured by a haemocytometer at each designated day, revealing increased proliferation of S16 cells at day 6 of the experiment, as compared to C9 cells (*P < 0.05). (E) Expression levels of the STAT3 target, *NR4A3,* were examined in GC cancer cell lines and transfectants and compared to those of MKN28 cells, set to 1.0 as a positive control (***P < 0.0001). Each bar represents mean± SD of duplicate experiments.

To examine the effect of STAT3 on epigenetic alteration of its downstream targets, we first established a constitutively activated STAT3 gastric cancer (GC) model using MKN28 human GC cell lines^24^. Either an empty vector control or vector containing *Stat3c*, a constitutively activated mouse Stat3 mutant^30^, were then transfected into MKN28 GC cells. After several rounds of selection, *Stat3c* (S16) stable transfectants, and empty vector control (C9) cells, were obtained (Fig. 1A).

The successful stable transfection of *Stat3c* in S16 GC cells was confirmed by increased expression of total STAT3 and the presence of FLAG (Fig. 1B). Likewise, hyperphosphorylation of Stat3 was observed in S16, but not in C9 vector control or MKN28 GC parental cells, suggesting that STAT3 signaling is constitutively activated in S16 cells. This phenomenon can also be observed in AGS GC cells, in which constitutive activation of STAT3 signaling has previously been reported^20^,^24^.

To examine whether Stat3 was functionally active in S16 cells, we examined the expression of the STAT3 upregulated targets, *MMP7*^31^, *Bcl-2*^32^ and downregulated target, *Mac-2bp*^33^ in S16 and C9 cells. Upregulation of *MMP7* and *Bcl-2* while down-regulation of *Mac-2BP* mRNA was observed in S16 cells as compared to C9 cells (Fig. 1C). Interestingly, upregulation of *MMP7* was also observed in AGS (Supplementary Figure S1). In addition, S16 cells also showed a slight but significant increase in cell growth (Fig. 1D). Taken together, we successfully established a stable clone with constitutively active Stat3 signaling, via gene overexpression, in MKN28 GC cells.

### Combined expression microarray and bioinformatic analyses identify NR4A3 as an epigenetically silenced STAT3 target

To identify genes differentially expressed after Stat3 constitutive activation, gene expression microarray analysis was performed to compare the expression profiles of S16 and C9 cells (Fig. 1A). To further identify differentially expressed genes that were regulated by STAT3, we performed bioinformatic analyses for genome-wide CpG island promoters containing STAT3-binding sites (Fig. 1A, Supplementary Table S3). Combining the results of the expression arrays and bioinformatic analyses of S16 cells, we found 49 upregulated genes with STAT3-binding sites in their promoter CpG islands, and 23 downregulated genes, respectively (Fig. 1A).

Our previous ovarian cancer study demonstrated that aberrant TGF-β/SMAD4 signaling may lead to epigenetic silencing of its downstream targets^29^,^34^. To identify if aberrant JAK/STAT signaling plays a similar role in GC, we focused on downregulated genes in S16 cells. One downregulated target, *NR4A3* (nuclear receptor subfamily 4, group A, member 3), was chosen for further investigation. NR4A3 was previously found to function as a tumor suppressor in acute myeloid leukemia cells^35^. To confirm our result, RT-PCR showed that *NR4A3* mRNA was downregulated in S16 cells having constitutively activated STAT3, as compared to C9 or MKN28 cells (Fig. 1E). It was interesting to note that decreased expression of *NR4A3* was also observed in AGS GC cells, in which STAT3 was also constitutively activated (Fig. 1E).

### *NR4A3* is epigenetically silenced by promoter DNA methylation in gastric cancer

**Figure 2:**
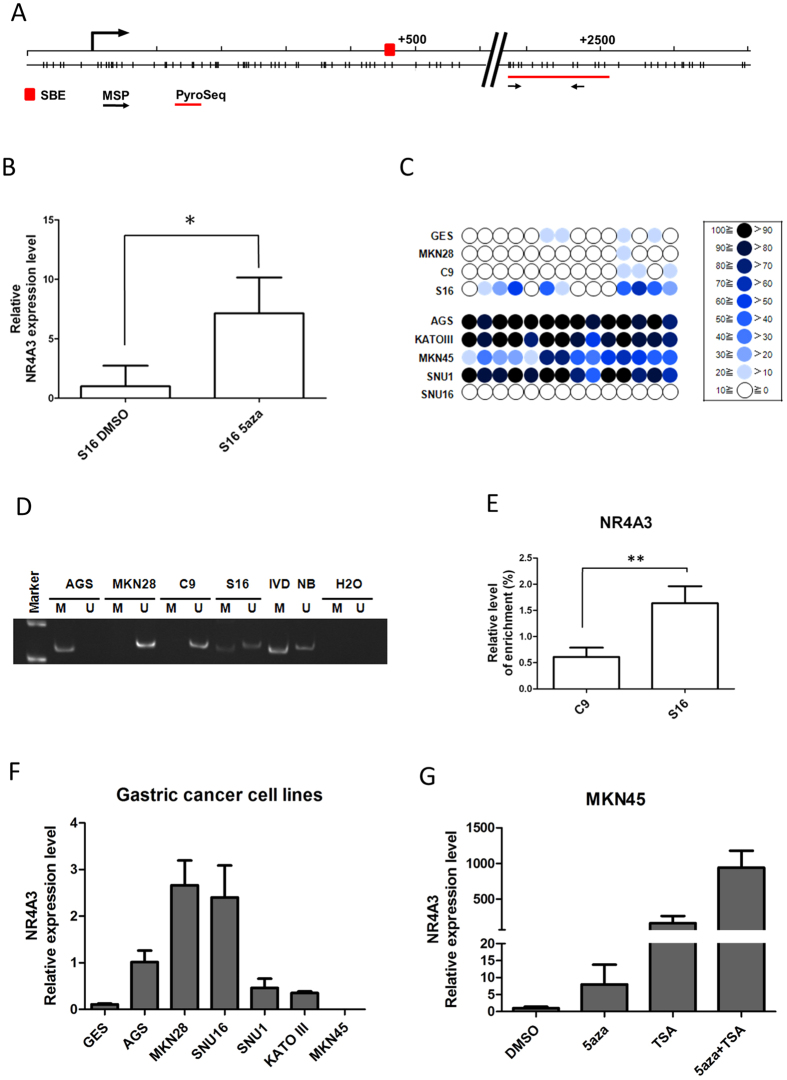
*NR4A3* is repressed by promoter DNA methylation in S16 and other gastric cancer cells. (**A**) Schematic diagram showing the genomic map of the *NR4A3* promoter, with corresponding locations of CpG sites and a putative STAT3-binding element (SBE, red box). CpG sites interrogated by bisulphite prosequencing (PyroSeq) and MSP are indicated by the red line and black arrows, respectively. (**B**) *NR4A3* expression in S16 cells, following DNA demethylation treatment with 0.5 μM 5-aza-2′-deoxycytidine (5-aza) or DMSO control, was examined by qRT-PCR. As depicted, 5-aza treatment significantly restored *NR4A3* expression (*P < 0.05). (**C**) Bisulphite pyrosequencing was performed to quantitatively examine the methylation levels of 14 CpG sites within the *NR4A3* promoter CpG island in C9, S16, and other gastric cancer (GC) cell lines. The percent methylation of each CpG site (circle) is indicated by the intensity of the blue color. *NR4A3* promoter methylation was also examined by (**D**) methylation-specific PCR (MSP) and (**E**) methyl-binding protein DNA capture (MBDcap) coupled to PCR. For MSP, bisulphite-modified DNA was PCR-amplified using specific primers. “M” and “U” indicate the presence of methylated and unmethylated alleles, respectively. IVD (*in vitro* methylated DNA) was a positive control for methylation and NB (normal blood) was a negative control for methylation. Water (H_2_O) was used as a negative control for PCR. In MBDcap-PCR, methylated DNA fragments were immunoprecipitated by MBD protein followed by qPCR (**P < 0.001). (**F**) Relative expression of *NR4A3* in GC lines and (**G**) 5-aza-treated MKN45 GC cells, as determined by qRT-PCR. Each bar represents mean± SD of dupliate experiments.

To investigate whether epigenetic modifications contribute to *NR4A3* downregulation in S16 GC cells, *NR4A3* mRNA expression was analyzed after demethylation treatment with the DNA methyltransferase inhibitor, 5-aza-2′-deoxyglucose (5-aza). 5-aza derepressed *NR4A3* expression, as validated by RT-PCR (Fig. 2B). Bisulphite pyrosequencing (Fig. 2C), and methylation-specific PCR (Fig. 2D) also clearly demonstrated *NR4A3* promoter hypermethylation in its CpG island “shore”^36^ (Fig. 2A) in S16, but neither in C9 nor the parental MKN28 cells. To exclude the possibility of incomplete bisulphite conversion, or the presence of 5-hydroxymethylcytosine (5hmC)^37^. we performed MBD (methyl-binding domain) immunoprecipitation^38^, followed by PCR, showing that methylated cytosine was enriched in the promoters of S16, but not C9, cells (Fig. 2E).

We then investigated possible epigenetic silencing of *NR4A3* in other GC cell lines. Except for immortalized gastric epithelial (GES) and SNU16 GC cells, promoter hypermethylation was found in all other GC cell lines (Fig. 2C). Results from RT-PCR were consistent with promoter DNA methylation, except for GES cells, in which the *NR4A3* promoter was devoid of methylation (Fig. 2F). Robust restoration of *NR4A3* expression was observed in MKN45 GC cells treated with 5aza or the histone deacetylase (HDAC) inhibitor trichostatin A (TSA), separately or in combination (Fig. 2G). Taken together, our results suggested that *NR4A3* is epigenetically silenced by promoter hypermethylation in GC cell lines.

### Activation of STAT3 suppresses *NR4A3* expression by promoter hypermethylation

**Figure 3:**
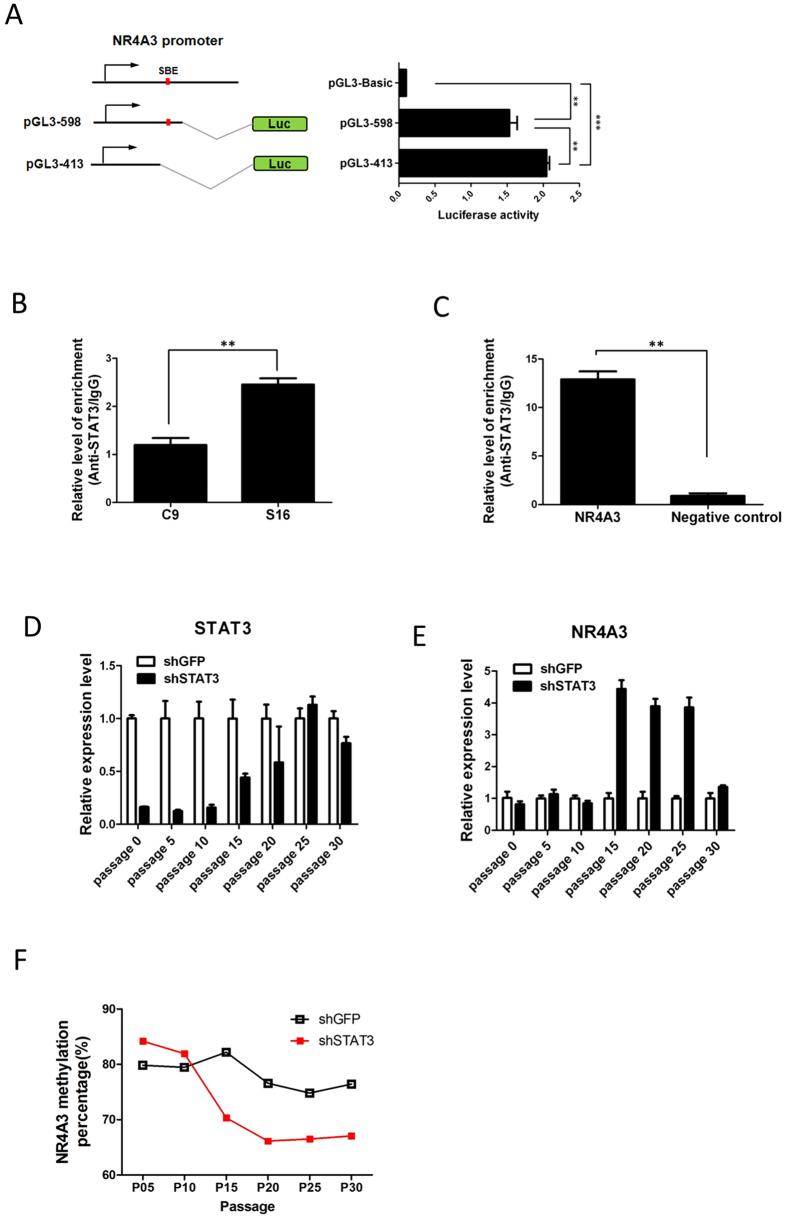
Binding of STAT3 represses *NR4A3* expression by promoter methylation in AGS GC cells. (**A**) *NR4A3* promoter regions with (598-bp fragment) or without (413-bp fragment) a putative STAT3 binding site were cloned into pGL3 luciferase-expressing vectors and transfected into AGS GC cells. 24 hours after transfection, luciferase reporter activities were determined. The promoter fragment lacking a STAT3-binding site (pGL3-413) showed significantly (**P < 0.001; ***P < 0.0001) higher luciferase activity than that with a STAT3-binding site (pGL3-598). (**B**) ChIP-PCR showing that STAT3 binding was significantly enriched in the *NR4A3* promoter of S16 cells, as compared to C9 cells. (**C**) As compared to an unrelated control (*GAPDH* promoter), STAT3 binding was significantly enriched in the *NR4A3* promoter in AGS cells. Expression of (**D**) *STAT3* and (**E**) *NR4A3*, in AGS cells, following long-term depletion of STAT3 by lentiviral shRNA knockdown, at each indicated cell passage number. (**F**) *NR4A3* promoter methylation, at each indicated passage number, following long-term STAT3 depletion, as determined by bisulphite pyrosequencing. shGFP, negative control.

Having demonstrated that *NR4A3* was epigenetically silenced in GC, we next investigated whether *NR4A3* expression was directly regulated by STAT3. Luciferase reporter assays of the *NR4A3* promoter, with or without its putative STAT3-binding site, were performed in AGS GC cells (Fig. 3A). Robust luciferase activity was observed in the promoter containing a putative STAT3-binding site (598 bp fragment). As expected, promoters without the putative STAT3-binding site (413 bp fragment) showed significantly upregulated luciferase activity (Fig. 3A). Surprisingly, transient STAT3 knockdown or treatment with JAK inhibitor, AG490 did not result in significant changes in luciferase activity (Supplementary Figure S2). These results suggest that other co-repressors may be involved in *NR4A3* suppression in a long term manner.

As compared to C9 cells, ChIP-PCR showed that STAT3 binding to the *NR4A3* promoter was significantly higher in S16 cells, with lower *NR4A3* expression (Fig. 3B). Also as expected, STAT3 binding was observed in the *NR4A3* promoter, but not in a negative control region (GAPDH), in AGS GC cells, with similarly low *NR4A3* expression (Fig. 3C).

Since the above experiments suggested that STAT3 might suppress *NR4A3* expression by promoter methylation in GC, we examined *NR4A3* expression in AGS cells depleted of STAT3. Lentiviral knockdown, in AGS cells, resulted in *STAT3* downregulation for at least 15 passages (Fig. 3D). However, *STAT3* expression was gradually restored from passage 20 onward, probably due to methylation of the LTR-driven promoter of the lentiviral shRNA vector (i.e., “position effect variegation”)^39^. To our surprise, expression of *NR4A3* was not restored immediately, but only after 15 passages of STAT3 knockdown (Fig. 3E).

We then investigated *NR4A3* promoter methylation during the course of *STAT3* knockdown. While *NR4A3* promoter methylation in the control cells remained high, it began to decrease at passage 15 in STAT3-knockdown cells, and remained more of less the same until the end of the experiments (Fig. 3F). Such multiple passage number dependence of *NR4A3* promoter demethylation resembles the progressive loss of DNA methylation (i.e. passive DNA demethylation) through inhibition of DNMT1, by 5-aza incorporation, during DNA replication^40^. Taken together, these results strongly suggest that STAT3 expression and activation can suppress *NR4A3* expression, in a long-term and stable manner through promoter DNA hypermethylation.

### Ectopic expression of *NR4A3* inhibits tumor growth *in vitro* and *in vivo*

**Figure 4:**
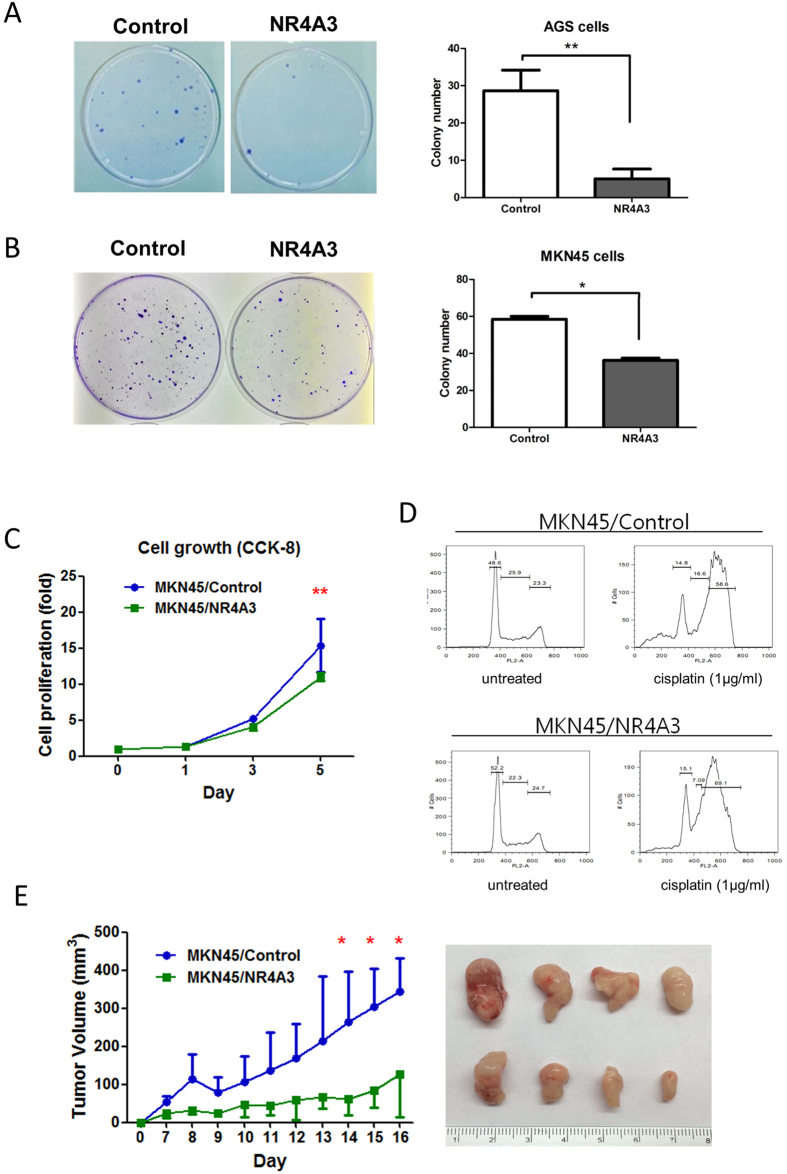
Ectopic expression of *NR4A3* inhibits tumor growth *in vitro* and *in vivo* in a xenograft mouse model. Ectopic expression of *NR4A3* inhibited tumor growth, as determined by colony formation assay. AGS or MKN45 GC cells transfected with empty or *NR4A3* expression vector were selected for further experiments (Supplementary Figure S1). (**A**) AGS or (**B**) MKN45 GC cells overexpressing *NR4A3* had significantly fewer colonies than the control. Right panel, quantitative analysis of the colony formation assay. (**C**) Cell growth of MKN45 GC cells, with or without NR4A3 expression, was determined by cell counting. Ectopic expression of *NR4A3* significantly inhibited cell growth in MKN45 GC cells. (**D**) Flow cytometry analysis of MKN45 GC cells with or without cisplatin (1 μg/ml) for 24 hr. DNA fluorescence histogram shows that *NR4A3* overexpression enhanced G2/M arrest in cisplatin-treated cells (please also refer to Supplementary Figure S4). (**E**) Effect of *NR4A3* overexpression on *in vivo* tumor growth in a nude mouse model. MKN45 cells stably transfected with *NR4A3* or empty vector (control) were injected subcutaneously into both flanks of athymic nude mice. Tumor volumes were measured daily. Representative examples of tumors formed in nude mice are also shown (right panel). **P < 0.01; *P < 0.05.

Given that *NR4A3* is a tumor suppressor in acute myelocytic leukemia^35^, our results demonstrated that *NR4A3* is epigenetically silenced in GC. Consequently, we investigated the functional role of NR4A3 in GC cell lines. Ectopic expression of *NR4A3* (Supplementary Figure S3) inhibited growth of AGS (Fig. 4A) and MKN45 GC cells (Fig. 4B,C, and Supplementary Figure S4). The lack of a significant sub-G1 population in *NR4A3*-overexpressing cells suggested that NR4A3 might not act through conventional apoptotic pathways (Fig. 4D). However, expression of *NR4A3* significantly sensitized MKN45 GC cells to cisplatin (Supplementary Figure S5) by restoring G2/M arrest (Fig. 4D and Supplementary Figure S6). Finally, nude mice subcutaneously injected with *NR4A3*-overexpressing MKN45 cells grew tumors of significantly less volume, as compared to mice injected with MKN45 cells with empty vector only (Fig. 4E). Taken together, these *in vitro* and *in vivo* results indicate *NR4A3* to be a potential tumor suppressor in gastric cancer.

### Epigenetic suppression of NR4A3 is associated with poor prognosis in gastric cancer patient samples

**Figure 5:**
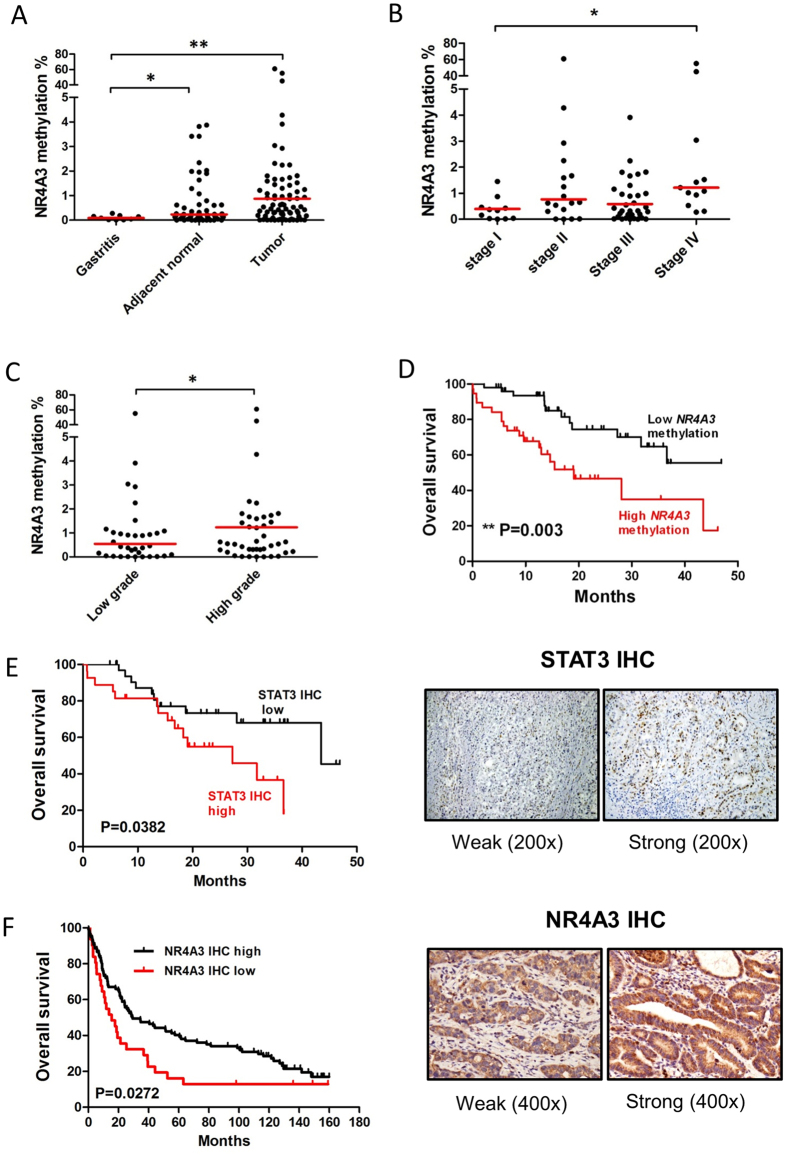
*NR4A3* promoter hypermethylation and increased STAT3 activity correlate with poor survival in gastric cancer patients. Quantitative real time MSP (qMSP) was performed to determine *NR4A3* methylation levels in gastritis (n = 9) and primary gastric cancer (GC) patient samples (n = 88). *NR4A3* methylation was significantly higher in (**A**) GC tumor samples and matched adjacent normal tissues, as compared to gastritis. *NR4A3* methylation in tumor samples also showed a progressive increase with (**B**) stage and (**C**) grade. (**D**) Kaplan-Meier analysis of *NR4A3* methylation in tumor tissues for overall survival of gastric cancer patients. GC patients with higher *NR4A3* methylation demonstrated shorter overall survival than patients with lower methylation (log-rank test, **P = 0.003). (**E**) Kaplan-Meier analysis also showed that patients with higher STAT3 nuclear staining (“STAT3 IHC high,” red line, vs. “STAT3 IHC low, black line) had significantly (P = 0.0382) shorter overall survival than patients with lower STAT3 nuclear staining (representative STAT3 IHC images shown in right panel). (**F**) NR4A3 IHC, as performed on another independent cohort of 128 GC patient tumors, using tissue microarrays. Similar to high STAT3 IHC, Kaplan-Meier analysis also showed that patients with lower NR4A3 staining (“NR4A3 IHC low,” red line, vs. “NR4AC IHC low,” black lines) had shorter (P = 0.0272) overall survival than those with higher *NR4A3* staining. Representative NR4A3 IHC images are shown in the right panel.

The above experiments demonstrated *NR4A3* as a potential tumor suppressor that is epigenetically silenced by promoter methylation, upon STAT3 activation. To investigate the clinical significance of *NR4A3* methylation in gastric carcinogenesis, we performed quantitative methylation-specific PCR (qMSP) to analyze *NR4A3* methylation in human gastritis and primary gastric tumor samples (Supplementary Table S1). *NR4A3* methylation levels in tumor tissues were significantly higher than those found in samples from adjacent normal or gastritic tissue specimens (Fig. 5A). *NR4A3* methylation also progressively increased according to advancement in tumor stage (Fig. 5B), grade (Fig. 5C), and lymph node metastasis (Supplementary Table S4). Patients with higher *NR4A3* methylation had significantly shorter survival than those with lower *NR4A3* methylation (Fig. 5D, Table 1). Interestingly, patients with high STAT3 activation also had shorter survival (Fig. 5E, Table 1), while Cox regression analysis demonstrated that *NR4A3* methylation could serve as an independent prognostic factor for overall survival (Table 1, multivariate analysis). We also analyzed the relationship between STAT3 activation and *NR4A3* methylation in this sample cohort. Intriguingly, STAT3 activation significantly associated only with *NR4A3* methylation in early-stage patient samples (Supplementary Table S5).

As *NR4A3* methylation associated with GC patient shorter survival, we performed immunohistochemistry (IHC) to investigate NR4A3 expression in tissue microarrays of another independent cohort containing 128 GC tumor samples (Supplementary Table S1). In this second cohort, patients with low *NR4A3* expression did not associate with any clinical parameters (Supplementary Table S6), except shorter overall survival (Fig. 5F, Supplementary Table S7).

## Discussion

DNA methylation, an epigenetic modification, is a genome-wide biological phenomenon which plays an essential role in gene dosage regulation in diverse cellular processes, including genomic imprinting and X chromosome inactivation, and also governs cell fate commitment. Several studies have shown that dysregulated DNA methylation participates in the development of numerous disease states, including human cancers^41^. However, how DNA methylation goes awry in human cancer is not fully understood.

Several studies have already demonstrated that activation of JAK/STAT signaling is crucial to gastric carcinogenesis with particular regard to *H. pylori*-infected tissues^8^,^17^,^42^; however, its role in epigenetic silencing of its target genes has not been fully explored. Since we previously demonstrated that aberrant TGF-β/SMAD4 signaling in ovarian cancer led to epigenetic silencing of its downstream targets^29^,^34^, we further examined whether such “signaling-mediated epigenetic silencing”^22^ exists in gastric cancer (GC), through aberrant activation of JAK/STAT signaling. By establishing hyperactivated STAT3 signaling in MKN28 GC cells, we showed that epigenetic silencing of *NR4A3*, a nuclear receptor and putative STAT3 target, could be derepressed. Interestingly, epigenetically silenced *NR4A3* could also be observed in AGS GC cells having constitutive, endogenous activation of STAT3 signaling. However, it is noteworthy to point out that *NR4A3* methylation was not observed in immortalized gastric epithelial GES cells, which do not express *NR4A3*. Thus, the role of other epigenetic modifications, such as histone acetylation/methylation, in regulating *NR4A3* transcription, cannot be excluded.

Our results from promoter luciferase assays and ChIP-PCR suggest that STAT3 might act as a transcriptional repressor of *NR4A3*. Importantly, depletion of STAT3 in AGS GC cells resulted in *NR4A3* promoter demethylation and re-expression. It is noteworthy that transient STAT3 knockdown or treatment with JAK inhibitor, AG490 did not result in a significant increase of promoter luciferase activity in AGS cells. While derepression and presumably, demethylation of *NR4A3* could only be achieved after long-term depletion of STAT3 (≥15 passages). Taken together, it is thus suggested that *NR4A3* repression is established in a stable, STAT3 concentration-dependent manner, in AGS cells. Repression of *NR4A3* was rapidly restored after *STAT3* re-expression, indicating that STAT3 may be a key initiator in the transcriptional repression of *NR4A3*. However, involvement of other co-repressors cannot be excluded.

Several groups have previously demonstrated that aberrant activation of transcription factors can induce epigenetic silencing of their target genes. For example, Di Croce *et al*. discovered that activation of the oncogenic fusion protein, PML-RARα, resulted in promoter hypermethylation and transcriptional repression of its downstream target gene, *RARβ*, by recruitment of a DNA methyltransferase (DNMT)^43^. Wasik and colleagues further demonstrated that malignant T lymphocytes, with persistent activation of STAT3, could directly bind and recruit DNMT1, as well as histone deacetylase-1 (HDAC1), to the promoter of the tyrosine phosphatase gene *SHP1*, resulting in its promoter DNA hypermethylation and epigenetic silencing^44^. Our current study, however, is the first to demonstrate that activation of STAT3 could result in promoter hypermethylation and epigenetically silence another target, *NR4A3*, in gastric cancer. Thus, the involvement of certain corepressors, such as the Daxx^45^, in the recruitment of DNMT to the *NR4A3* promoter, deserves further investigation.

NR4A3, also known as NOR-1, is a nuclear receptor and transcription factor involved in various cellular, metabolic, and tumor suppressor processes^46^,^47^,^48^,^49^. Mullican *et al*., using *NR4A1*/*NR4A3* double-knockout mice, reported that loss of these two genes can result in the development of acute myelocytic leukemia (AML), due to uncontrolled expansion of myeloid progenitor cells^35^. Consistent with those results, our *in vitro* and *in vivo* functional studies also showed that overexpression of *NR4A3* inhibited tumor growth (Fig. 4). Moreover, GC patients with higher methylation or lower expression of *NR4A3* demonstrated significantly shorter overall survival, indicating a potential tumor suppressor role (Fig. 5C). Given that activation of STAT3 signaling resulted in epigenetic silencing of *NR4A3* in GC, we expected to observe a positive correlation between STAT3 activation and *NR4A3* methylation in our sample cohort. However, such a correlation could only be observed in early-stage tumor samples (Supplementary Table S5, P = 0.01). This phenomenon might be due to the fact that STAT3-mediated epigenetic silencing of *NR4A3* is initiated during early gastric carcinogenesis; however, this must be confirmed in a larger sample cohort.

Activation of STAT3 was previously found to associate with drug resistance in GC^50^, and derepression of *NR4A3* sensitized gastric cancer cells to cisplatin. In this regard, the shorter overall survival we observed in patients with *NR4A3* methylation may likely be due to chemo-resistance-related tumor recurrence. Thus, *NR4A3* methylation might be an effective prognostic marker for predicting therapeutic response.

Several studies has previously demonstrated that targeted inhibition of STAT3 could be a novel therapeutic strategy against GC^51^,^52^. Our study may provide a mechanistic reason such that inhibition of STAT3 may reverse the epigenome and thus restoring the expression of tumor suppressor genes. Combination of STAT3 inhibitor together with low dose epigenetic modifiers such as 5aza, might result in a more specific reversal of the epigenome and derepression of tumor suppressors, thus deserves further clinical trial in GC.

There are several limitations in our present study. First, as a retrospective study, selection bias may exist and cannot be totally avoided, even after multivariate analysis adjustment. Second, no association between *H. pylori* infection and methylation of *NR4A3* was found in this tumor sample cohort. This shortcoming may be attributed to the relatively small sample size of *H. pylori-*positive samples (n = 6). Therefore, prospective studies, with a more balanced sample-distribution, should be conducted to confirm any hypothesized associations.

Taken together, our results reveal that constitutive activation of JAK/STAT3 signaling can lead to transcriptional silencing of a STAT3 target, *NR4A3*, through aberrant promoter DNA methylation. Overexpression of *NR4A3* suppressed tumor growth *in vitro* and *in vivo*, strongly suggesting that it may be a novel tumor suppressor in GC, as increased *NR4A3* hypermethylation associated with shorter overall survival. In conclusion, *NR4A3* methylation may be able to serve as a diagnostic and prognostic biomarker for GC. The therapeutic potential of targeted inhibition of STAT3 in GC deserves further investigation.

## Methods

### Study subjects and tissue samples collection

Genomic DNA from 88 gastric tumor and matched adjacent normal samples were collected from the Changhua Christian Hospital, Chang-Hua, Taiwan (clinical-pathological data summarized in Supplementary Table S1). For tissue microarrays, another independent cohort, containing 128 gastric tumor specimens, was also procured from the same hospital. For a negative control, genomic DNA from nine non-cancer gastritis patients was collected at the Prince of Wales Hospital, Shatin, Hong Kong. All human subject assessments were approved by the Institutional Review Board (IRB) of Changhua Christian Hospital, Chang-Hua, Taiwan, and the Joint Chinese University of Hong Kong-New Territories East Cluster Clinical Research Ethics Committee. The study was carried out in strict accordance with approved guidelines. Informed consent was obtained from all participants.

### Cell culture and epigenetic treatment

Six gastric cancer cell lines (AGS, MKN28, MKN45, KATOIII, SNU1, and SNU16) and an immortalized gastric epithelial cell line, GES (a kind gift from Dr. Jun Yu, The Chinese University of Hong Kong, Hong Kong), were maintained in RPMI-1640 medium (Invitrogen, Carlsbad, CA, USA). The transformed human embryonic kidney cell line (HEK293T) was maintained in DMEM (Invitrogen). All the culture media used was supplemented with 10% fetal bovine serum (FBS, Invitrogen), 50 units/ml of penicillin/streptomycin (Invitrogen), and cells were incubated at 37 °C under a humidified atmosphere containing 5% CO_2_. For DNA demethylation treatment, cells were seeded in 60-mm plates and treated with 0.5 μM 5′-aza-2′-deoxycytidine (5aza, Sigma, St. Louis, MO, USA) for 72 hr, with or without 0.5 μM of the histone deacetylase (HDAC) inhibitor trichostatin A (TSA, Sigma) for 12 hr. Culture media and drugs were replenished every 24 hr. Following the various treatments, the cells were lysed and harvested for DNA or RNA analyses. Experiments were repeated twice.

### mRNA expression microarray

Gene expression microarray analysis was performed by Welgene Biotech (Taipei, Taiwan), using Agilent (Santa Clara, CA, USA) Human Whole Genome 44 K expression microarrays, and expression-varied (1.5-fold up- or down-regulated) genes were chosen for further assessment. The microarray data has been deposited in the Gene Expression Omnibus database (accession number: GSE78714).

### Real-time quantitative methylation-specific PCR (qMSP)

For measuring DNA methylation, DNA was bisulphite-modified (resulting in deamination of unmethylated cytosine to uracil) using an EZ DNA methylation^TM^ kit (ZYMO Research, Irvine, CA, USA), according to the manufacturer’s protocol, and subjected to quantitative methylation-specific PCR (qMSP), as previously described^53^. PCR qMSP amplification was performed using the StepOne Real-Time PCR instrument (Applied Biosystems, Carlsbad, CA, USA), with ACTB used to normalize the input DNA. The absolute amount of methylated *NR4A3* was deter ed by the threshold PCR cycle number (Ct), for each sample, using a standard curve generated by qMSP of an SssI-treated cloned DNA fragment. The relative percentage of *NR4A3* methylation was calculated as the NR4A3 vs ACTB ratio for each sample, divided by the same ratio for SssI-treated sperm DNA (positive DNA methylation control, Millipore, Billerica, MA, USA) and multiplied by 100. Experiments were repeated twice.

### Bisulphite pyrosequencing

Bisulphite pyrosequencing was performed as described previously^54^. In brief, bisulphite-modified DNA was subjected to PCR amplification, using a tailed reverse primer and a biotin-labeled universal primer (Supplementary Table S2). PCR and sequencing primers, for a 194-bp fragment (+2338 to +2532) of the *NR4A3* promoter CpG island “shore”^36^, were designed using the PyroMark Assay Design 2.0 instrument (Qiagen, Hercules, CA, USA), and pyrosequencing performed using PyroMark Q24 (Qiagen) and Pyro Gold Reagents (Qiagen), according to the manufacturer’s protocol. Methylation levels of 14 CpG sites, within the 194-bp sequence, were measured, and methylation percentages for each cytosine were determined by their fluorescence intensities divided by the sum total fluorescence intensities of cytosines and thymines (converted from uracil by PCR), at each CpG site.

### Cell cycle analysis

MKN45/control or MKN45/*NR4A3* (S16) GC cells were harvested, washed with cold PBS, fixed with 100% cold methanol, and incubated overnight at 4 °C. The cells were subsequently treated with 50 μg/mL RNaseA (Invitrogen) and 40 μg/mL propidium iodide (Sigma-Aldrich) for 30 min. DNA content was analyzed using a FACScan flow cytometer (Becton Dickinson, Franklin Lakes, NJ, USA), with the cell cycle percentages of the cells calculated using ModFit software (Verity Software House, Topsham, MA, USA). Experiments were repeated twice.

### Immunohistochemical (IHC) analysis

Paraffin-embedded GC tissue samples from the above-mentioned patients were obtained from the Department of Pathology at the Changhua Christian Hospital, following Institutional Review Board approval. Tissue sections were dewaxed in xylene and rehydrated in alcohol. Antigen retrieval was performed by heating each section at 100 °C for 20 min in 10 mM sodium citrate buffer (pH 6.0). IHC followed a standard protocol using an anti-STAT3 monoclonal antibody (1:100, Cell Signaling, Danvers, MA, USA) and an anti-NR4A3 antibody (Cell Signaling), using the Novolink^TM^ Min Polymer Detection System (Leica, Wetzlar, Germany). IHC scores were calculated using the formula: intensity x percentage of cells showing positive staining. The STAT3 percent activation score was calculated using the formula: (nuclear IHC score)/(nuclear IHC score + cytosolic IHC score). The results were then assessed by two pathologists independently (CYL and KTY).

### *In vivo* tumorigenicity assay

To investigate the effect of *NR4A3* on GC growth *in vivo,* 1×10^7^ of MKN45/control or MKN45/*NR4A3* (S16) cells were injected subcutaneously into the flanks of 5-week-old female nude mice (BALB/cByJNarl, n = 4), obtained from the National Laboratory Animal Center, Taiwan. To reduce the number of animals used, the control and experimental groups used both sides of the same mouse. Tumor size was measured daily with calipers as length (L) and width (W), and tumor volume calculated using the formula (L x W^2^/2). At the end of the experiment, all mice were sacrificed by cervical dislocation. All mice were kept under specific pathogen-free conditions using a laminar airflow rack, with free access to sterilized food and autoclaved water. All experiments were approved by the Animal Experimentation Ethics Committee of National Chung Cheng University, Taiwan. This study was performed in accordance with the approved guidelines and regulations of National Chung Cheng University.

### Bioinformatic and statistical analysis

Bioinformatic analysis was performed to identify predicted STAT3-binding sites using the UCSC table browser (https://genome.ucsc.edu/cgi-bin/hgTables). Statistical significance was determined using GraphPad Prism Version 5.0 software packages for Windows (GraphPad Software, La Jolla, CA, USA). The Student’s *t* test or the Mann–Whitney U test was used to compare parameters of different groups. Overall survival (OS) was assessed by Kaplan-Meier analysis using log-rank test. Univariate and multivariate survival analysis was determined using a Cox proportional hazards model. Overall survival was defined as the duration from day of diagnosis to death. A DNA methylation level of *NR4A3* at 1% (methylation level in gastritis) was used as a cutoff, and for IHC scores for STAT3 and NR4A3, 50% was used as a cutoff. *P* < 0.05 was considered statistically significant.

Additional Materials and Methods can be found in supplementary information.

## Additional Information

**How to cite this article**: Yeh, C.-M. *et al*. Epigenetic silencing of the *NR4A3* tumor suppressor, by aberrant JAK/STAT signaling, predicts prognosis in gastric cancer. *Sci. Rep.* **6**, 31690; doi: 10.1038/srep31690 (2016).

## Supplementary Material

Supplementary Materials and Methods

Supplementary Table

Supplementary Figure

Supplementary Table S3

## Figures and Tables

**Table 1:** Hazard ratios for overall survival, based on predictive factors, in 88 gastric cancer samples.

	HR(95% CI); P value	
	Univariate analysis	Multivariate analysis
Age		
<60 vs ≥60	1.214(0.494–2.986);0.673	NA^3^
HP status		
no vs yes	0.517(0.121–2.217);0.375	NA
Grade^1^		
Low vs high	0.833(0.409–1.697);0.616	NA
Stage^2^		
low vs high	4.677(1.631–13.409);<**0.001**	4.402(0.927–17.619);0.063
Lymph node metastasis		
no vs yes	4.552(1.084–19.126);<**0.05**	2.063(0.186–22.878);0.555
STAT3 IHC		
inactive vs active	2.380(1.022–5.544);<**0.05**	1.921(0.881–4.188);0.101
NR4A3 methylation		
low vs high	2.878(1.379–6.006);<**0.005**	2.375(1.105–5.103);**<0.05**

^1^Pathologiocal grading, low: G1-2; high: G3.

^2^Staging, low:pT1-2; high:pT3-4.

^3^NA: not available.
