# Supplementary material for: Epigenetic silencing of the NR4A3 tumor suppressor, by aberrant JAK/STAT signaling, predicts prognosis in gastric cancer
Source: Sci Rep. 2016 Aug 16;6:31690. doi: 10.1038/srep31690 (PMC4985659; doi:10.1038/srep31690)
Supplement: Supplementary Materials and Methods [file srep31690-s1.pdf]

Supplementary Materials and Methods

Title of Manuscript:

Epigenetic silencing of the *NR4A3* tumor suppressor, by aberrant JAK/STAT signaling, predicts prognosis in gastric cancer

Authors:

Chung-Min Yeh, Liang-Yu Chang, Shu-Hui Lin, Jian-Liang Chou, Hsiao-Yen Hsieh, Li-Han Zeng, Sheng-Yu Chuang , Hsiao-Wen Wang, Claudia Dittner, Cheng-Yu Lin, Jora M.J. Lin, Yao-Ting Huang, Enders K.W. Ng, Alfred S.L. Cheng, Shu-Fen Wu, Jiayuh Lin, Kun-Tu Yeh and Michael W.Y. Chan

Plasmid constructs

The plasmid pRC/CMV-Stat3c, a constitutively activated STAT3 mutant (a gift from James Darnell, Rockefeller University, NY), was subcloned into pcDNA3.1 for transfection. The NR4A3 coding sequence was amplified by PCR using specific primers using total cDNA from MKN28 cells. The PCR products were then ligated to the yT&A cloning vector (Yeastern Biotech, Taiwan) for sequencing, and finally, subcloned into pcDNA3.1 or pcDNA4/TO for transfection. Promoter regions, with or without putative STAT3 binding element of the NR4A3 putative promoter, was amplified by PCR with specific primers covering the region from -59 to +354bp or -59 to +539, using MKN28 cell genomic DNA. The PCR products were ligated into yT&A vector (Yeastern Biotech) for sequencing and finally, subcloned into pGL3-Basic (Promega, Madison, WI) for promoter luciferase reporter assays. Mammalian expression vectors were transfected into gastric cancer cell lines using Lipofectamine 2000 transfection reagent (Invitrogen), according to the manufacturer's protocol.

Transfected cells were cultivated with fresh culture medium containing 400 μg/ml Geneticin® (G418, Invitrogen), which was replaced every 3 days. Single colonies were then selected for establishment of stable clones. All primer sequences are listed in Supplemental Table S2.

Lentivirus-based RNAi-induced target gene permanent knockdown

An shRNA targeted to STAT3 was acquired from the National RNAi Core Facility Platform at the Institute of Molecular Biology/Genomic Research Center, Academia Sinica, Taiwan. Briefly, to prepare the shSTAT3 lentivirus, 293T cells were transfected with shRNA (TRCN0000051173 and TRCN0000303423), pMDG, and pCMVR 8.91 using ProFection Mammalian Transfection System (Promega). Infected cells were selected by incubating with puromycin (2 μg/mL, Sigma) for at least 2 days.

Reverse transcription and quantitative real-time polymerase chain reaction (q-PCR)

RNA extraction was performed using Trizol reagent (Invitrogen), according to the manufacturer’s protocol. Total cellular RNA (1 μg) was treated with Amplification Grade Deoxyribonuclease I (DNase I, Invitrogen), prior to reverse transcription using the SuperScriptTM III Reverse Transcription System (Invitrogen), according to the manufacturer's instructions. Primers were designed for MMP7, NR4A3, and STAT3 using primer expression software version 3.0 (Applied Biosystems, Foster City, CA). The 2×SYBR Green Real-time PCR Master Mix (Toyobo, Japan) and cDNA of all cell lines were subjected to real time PCR amplification by the StepOne Real-Time PCR system (Applied Biosystems). The relative gene expression level was determined by comparing the threshold cycle (Ct) of the test gene against the Ct of GAPDH in a given sample (i.e., the comparative Ct method). Experiments were repeated twice.

Protein extraction and Western blot

Cells were lysed with 100μl of PRO-PREP Protein Extraction Solution (iNtRON Biotechnology, Korea) and protein concentrations determined by Bio-Rad Protein Assay kit (Bio-Rad, Hercules, CA), according to the manufacturer's protocol. Samples and prestained markers were electrophoresed through 12.5% polyacrylamide gels, and then transferred to PVDF membranes, using the Mini Trans-Blot Electrophoretic Transfer Cell system (Bio-Rad, Hercules, CA) at 400 mA for about 90 minutes. After transfer, the membranes were washed with 5% non-fat milk in 1X TBST for 1 hour at room temperature, and then incubated overnight at 4°C with primary antibodies, rabbit anti-pSTAT3 (1:1000, Cell Signaling, Beverly, MA), rabbit anti-STAT3 (1:1000, Cell Signaling), mouse anti-FLAG (1:1000, Cell Signaling) or mouse anti-GAPDH antibodies (1:2000, Thermo Fisher Scientific, Rockford, IL) , as diluted with 5% non-fat milk in 1X TBST. The membranes were then washed 3 times with 1X TBST at room temperature , and incubated at room temperature for one hour, with secondary antibodies (Thermo Fisher, anti-mouse, 1:4000 or anti-rabbit 1:4000, diluted in 5% non-fat milk in 1X TBST), conjugated to horseradish peroxidase, followed by washing in 1X TBST for 3 times. The Immobilon Western Chemiluminescent HRP Substrate (Merck Millipore, Billerica, MA) was prepared by mixing equal volumes of HRP Substrate Luminol Reagent, as well as the HRP Substrate Peroxide Solution, added to the washed membranes, and chemiluminecent signals detected by X-ray film. Experiments were repeated twice.

Colony formation assay

AGS or MKN45 cells were transfected with pcDNA4/TO-NR4A3 or empty vector control, according to the manufacturer's protocol. Twenty-four hours after transfection,

cells were replated in triplicate and cultured for 3 weeks in RPMI 1640 containing 10% fetal bovine serum supplemented with 30 μg/ml ZeocinTM (Invitrogen), with media replaced every 3 days until observable colonies were obtained. The surviving colonies were then stained with 0.4% crystal violet (Sigma) in 50% methanol and counted.

Promoter luciferase reporter assay

pGL3-413, pGL3- 598, or pGL3-Basic empty vectors were cotransfected with Renilla luciferase vectors (pRL-TK) to AGS cells treated with control or JAK inhibitor, AG490 (Sigma) for 24 hours. Cells were then lysed and promoter luciferase activities measured using a Dual Luciferase Reporter Assay System (Promega, Madison, WI) on a luminometer (Turner Designs, Sunnyvale, CA). Experiments were repeated twice.

Cell viability assay

Cell viability was determined by the tetrazolium Cell Counting Kit-8 assay (Sigma), according to the manufacturer's instructions. Briefly, MKN45/control and MKN45/NR4A3 cancer cells were seeded into 96-well culture plates and cultured in medium containing 10% FBS with different concentration (0.04, 0.2, 1, 5 μg/ml) of cisplatin (Sigma) for 72 hr. After incubation, the medium was replaced with fresh medium and the cells were incubated with 5 mg/ml CCK8. Optical densities, at 450nm/655 nm, were then measured using an ELISA plate reader (Bio-Rad). Experiments were repeated twice.
