# Supplementary material for: Epigenetic silencing of the NR4A3 tumor suppressor, by aberrant JAK/STAT signaling, predicts prognosis in gastric cancer
Source: Sci Rep. 2016 Aug 16;6:31690. doi: 10.1038/srep31690 (PMC4985659; doi:10.1038/srep31690)
Supplement: Supplementary Table [file srep31690-s2.pdf]

Supplementary Tables

Title of Manuscript:

Epigenetic silencing of the *NR4A3* tumor suppressor, by aberrant JAK/STAT signaling, predicts prognosis in gastric cancer

Authors:

Chung-Min Yeh, Liang-Yu Chang, Shu-Hui Lin, Jian-Liang Chou, Hsiao-Yen Hsieh, Li-Han Zeng, Sheng-Yu Chuang , Hsiao-Wen Wang, Claudia Dittner, Cheng-Yu Lin, Jora M.J. Lin, Yao-Ting Huang, Enders K.W. Ng, Alfred S.L. Cheng, Shu-Fen Wu, Jiayuh Lin, Kun-Tu Yeh and Michael W.Y. Chan

Table S1. Summary of clinical-pathological data of gastric cancer samples

	Methylation analysis (n=88)	Tissue microarray (n=128)
**Age at diagnosis (years)**		
<60	21	33
≥ 60	67	95
**Stage**		
1	14	15
2	20	17
3	40	78
4	14	17
**Grade**[1]		
low	36	54
high	52	72
**Pathological Type**		
Diffuse	49	NA
Intestinal	30	
Mixed	9	
**HP status**		
-ve	66	NA
+ve	6	
**Lymph node metastasis**		
0	19	43
1	28	28
2	19	23
3	22	34

[1]Pathologiocal grading, low: G1-2; high: G3;

[2]NA: not available

Table S2. Primers used in this study

Primer name	Primer sequence (5' to 3')	Annealing Temp (°C)
**MSP & qMSP**		
NR4A3 MF	TTTTTCGCGAACGTTGGGC	60
NR4A3 MR	TAAAAACCTTTCAAACGAAATCG	
NR4A3 UF	GGTTTTTTTTGTGAATGTTGGGT	64
NR4A3 UR	CCCTAAAAACCTTTCAAACAAAATCA	
β-actin (*ACTB*) MF	TGGTGATGGAGGAGGTTTAGTAAGT	
β-actin (*ACTB*) MR	AACCAATAAAACCTACTCCTCCCTTAA	
**Bisulphite Pyrosequencing**		
NR4A3_Pyro-F	GGGTTGGGTTGGAGGAAAG	56
NR4A3_Pyro-R-UNIVR[1]	agctggacatcacctcccacaacgCCCCACCCYCCCCTTCAA	
Biotinylated-UNIVR	agctggacatcacctcccacaacg	
NR4A3_Seq	AGGTTGTGTGGGTTT	
**RT-PCR**		
NR4A3 RT F	ACAAGATAGCTTCAGACCAAATTCAAA	60
NR4A3 RT R	AGACACGACAAAAACAAGGATTAACTT	
MMP7 RT-F	GAACGCTGGACGGATGGTA	60
MMP7 RT-R	AGGTTGGATACATCACTGCATTAGG	
Bcl-2 RT-F	GTTCAAACAAGACGCCAACA	60
Bcl-2 RT-R	TGGGCCAGAGCTACATCTTT	
Mac-2bp RT-F	CTCACTGGCCGACTGCAA	60
Mac-2bp RT-R	GTGGGTGCTCCTGGTTTCAT	
GAPDH RT-F	CCCCTTCATTGACCTCAACTACAT	60
GAPDH RT-R	CGCTCCTGGAAGATGGTGA	
**ChIP-PCR**		
NR4A3 ChIP-F	GCCGCTGGGCTTGTACAC	60
NR4A3 ChIP-R	TGGCGGTTCCGTCTTTAGAG	
**Plasmid construction**		
**Promoter**		
NR4A3 P-F	TCACATTGACGTCTCGCATTC	58
NR4A3 P413-R	TCCTGGCTTGGGCTGTGA	
NR4A3 P598-R	GGTTCCGTCTTTAGAGCGAGTTT	
**cDNA**		
NR4A3 F-HindIII/Flag[2]	ACCaagcttATATGgactacaaggacgacgatgacaagggcCCCTGCGT CCAAGCCCAAT	
NR4A3 R-NotI[2]	ACTgcggccgcTTAGAAAGGTAGGGTGTCCAGGAAGAGCTT	

[1] Primer sequence of the 5'tailed universal primer (UNIVR) is shown as lower case;

[2] Enzyme cut site and FLAG epitope is shown as lower case.

Table S4. The association between *NR4A3* methylation and clinicopathological features in 88 gastric cancer patients.

	NR4A3 methylation %	P
Total (n=88)	43 (38/88)	
Age at diagnosis (years)		
<60	28.6 (6/21)	
≥ 60	47.8 (32/67)	0.096
Stage		
1	28.6 (4/14)	
2	45 (9/20)	
3	37.5 (15/40)	
4	71 (10/14)	0.097
Grade		
low	30.5 (11/36)	
high	51.9 (27/52)	0.038
Pathological Type		
Diffuse	38.7 (19/49)	
Intestinal	46.6 (14/30)	
Mixed	55.6 (5/9)	0.578
HP status		
-ve	40.9 (27/66)	
+ve	50 (3/6)	0.491
Lymph node metastasis		
0	42.1 (8/19)	
1	25 (7/28)	
2	47 (9/19)	
3	63.6 (14/22)	0.049
STAT3 nuclear score		
-ve	28.5 (10/35)	
+ve	51.8 (14/27)	0.054

Table S5. Association between STAT3 nuclear score and NR4A3 methylation in low-stage gastric cancer patients.

	NR4A3 methylation %	P
STAT3 nuclear score		
-ve	0 (0/8)	
+ve	58.3 (7/12)	0.01

Table S6. The association between NR4A3 IHC score and the clinicopathological features of 128 gastric cancer patients (tissue microarray)

	NR4A3 IHC score	P
Total (n=128)	75.8 (97/128)	
**Age at diagnosis (years)**		
<60	69.7(23/33)	
≥ 60	77.9(74/95)	0.236
**Stage**		
1	73.3 (11/15)	
2	76.5 (13/17)	
3	74.4 (58/78)	
4	82.4(14/17)	0.911
**Grade**[1]		
low	83.3 (45/54)	
high	69.4 (50/72)	0.055
**Lymph node metastasis**		
0	79.1 (34/43)	
1	89.3 (25/28)	
2	65.2(15/23)	
3	67.6 (23/34)	0.129

[1]Pathologiocal grading, low: G1-2; high: G3;

Table S7. Hazard ratios for overall survival according to predictive factors in 128 gastric cancer samples (tissue microarray)

	**HR(95% CI); P value**	
	**Univariate analysis**	**Multivariate analysis**
**Age**		
<60 vs ≥ 60	1.895 (1.161-3.092);**<0.05**	2.140(1.294-3.539);**<0.005**
**Grade**[1]		
Low vs high	1.421(0.954-2.116);0.084	NA
**Stage**[2]		
low vs high	1.769(1.098-2.848);**<0.05**	1.737(1.053-2.867);**<0.05**
**Lymph node metastasis** no vs yes	1.446(0.954-2.190);0.082	1.312(0.845-2.036);0.227
**NR4A3 IHC**		
low vs high	0.610(0.392-0.950);**<0.05**	0.558(0.354-0.878);**<0.05**

[1]Pathologiocal grading, low: G1-2; high: G3;

[2]Staging, low:pT1-2; high:pT3-4
