# Supplementary material for: Epigenetic silencing of the NR4A3 tumor suppressor, by aberrant JAK/STAT signaling, predicts prognosis in gastric cancer
Source: Sci Rep. 2016 Aug 16;6:31690. doi: 10.1038/srep31690 (PMC4985659; doi:10.1038/srep31690)
Supplement: Supplementary Figure [file srep31690-s3.pdf]

Supplementary Figures

Title of Manuscript:

Epigenetic silencing of the *NR4A3* tumor suppressor, by aberrant JAK/STAT signaling, predicts prognosis in gastric cancer

Authors:

Chung-Min Yeh, Liang-Yu Chang, Shu-Hui Lin, Jian-Liang Chou, Hsiao-Yen Hsieh, Li-Han Zeng, Sheng-Yu Chuang , Hsiao-Wen Wang, Claudia Dittner, Cheng-Yu Lin, Jora M.J. Lin, Yao-Ting Huang, Enders K.W. Ng, Alfred S.L. Cheng, Shu-Fen Wu, Jiayuh Lin, Kun-Tu Yeh and Michael W.Y. Chan

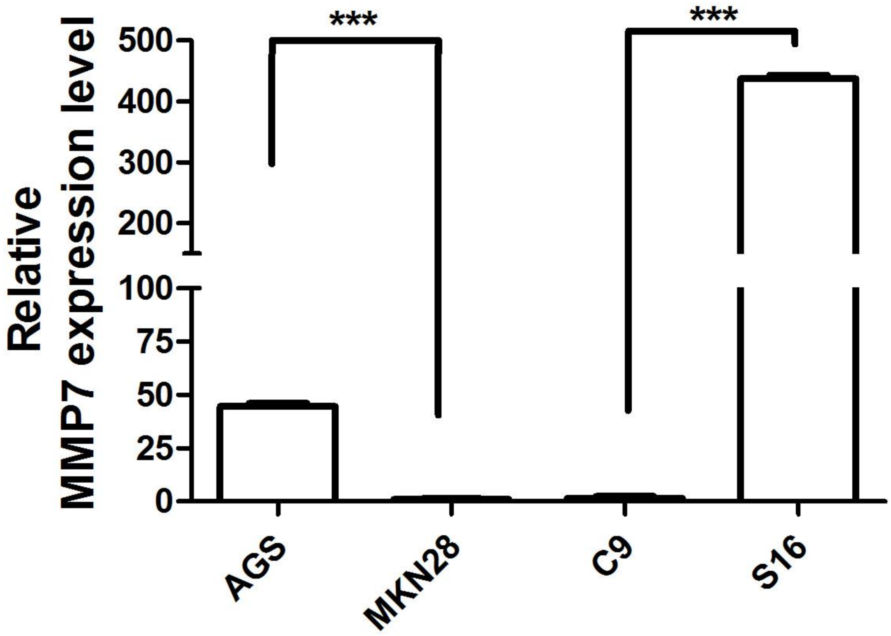

Figure S1. STAT3 activation in S16 cells was confirmed by the expression of *MMP7*, a STAT3 target, using qRT-PCR. Both S16 and AGS cells showed *MMP7* upregulation, compared to C9 control and MKN28 GC cells. (***, *P*<0.001).

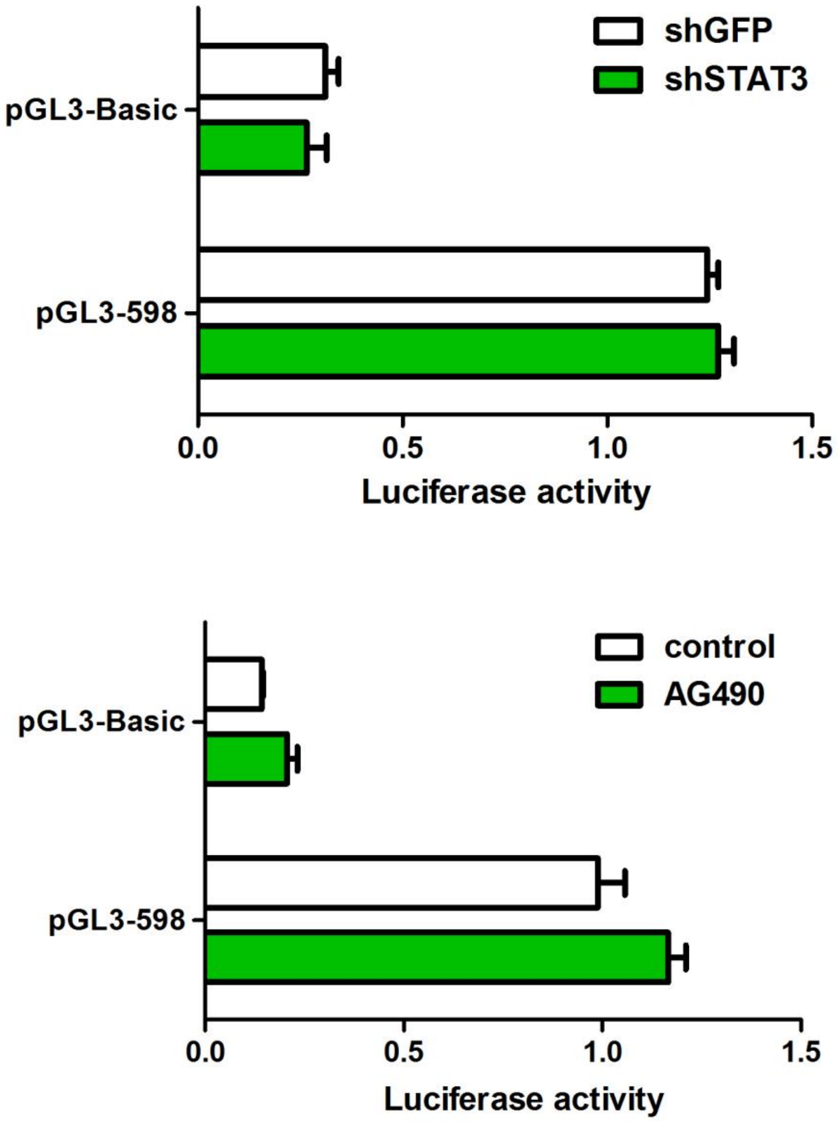

Figure S2. *NR4A3* promoter regions (598-bp fragment) with a putative STAT3 binding site were cloned into pGL3 luciferase-expressing vectors (pGL3-598) and transfected into AGS GC cells transiently knockdown with STAT3 (upper panel) or treated with 40 µm of JAK inhibitor, AG490 for. 24 hours (lower panel). 24 hours after transfection, luciferase reporter activities were determined. Transient STAT3 knock or treatment with AG490 did not result in a significant changes in luciferase activity in AGS GC cells.

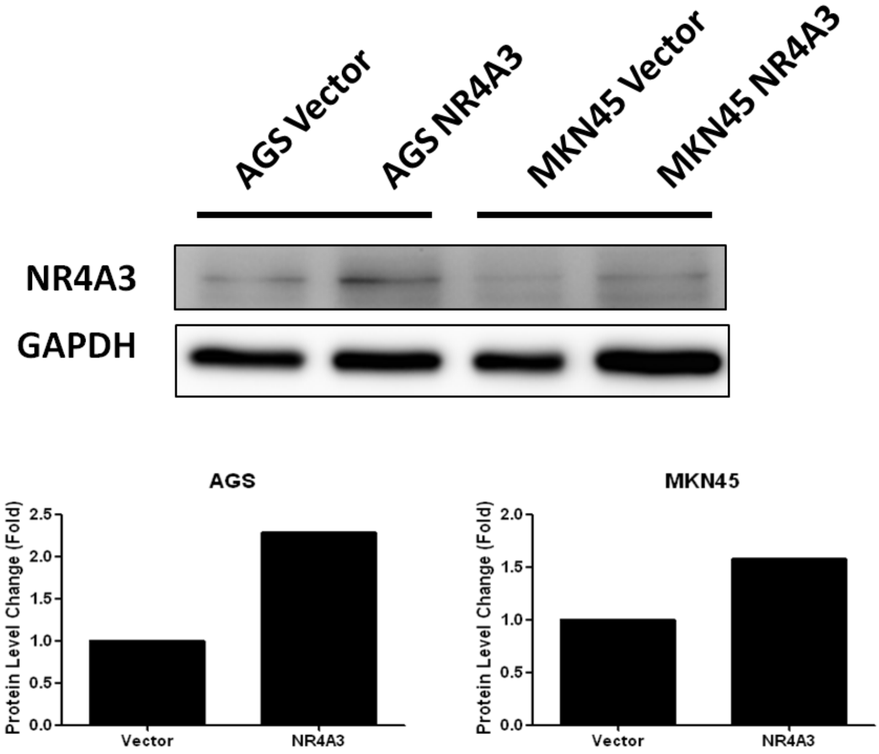

Figure S3. *NR4A3* expression of AGS or MKN45 gastric cancer cells transfected with plasmid overexpressing NR4A3 was determined by Western blotting analysis. GAPDH was used as a loading control. Lower panel showed the quantitative analysis of the Western blotting result as determined by ImageJ software (National Institutes of Health Bethesda, MD; http://rsb.info.nih.gov/ij/).

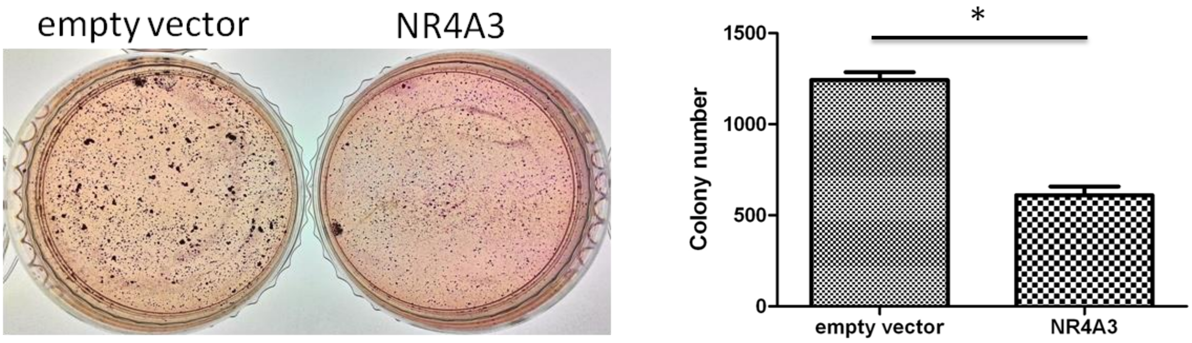

Figure S4. Ectopic expression of *NR4A3* inhibited tumor growth as determined by soft agar assays. MKN45 gastric cancer cells transfected with empty or NR4A3 expression vector were selected for soft agar growth assay. Cells stably overexpressing NR4A3 generated significantly fewer colonies than the controls. (Left panel) Quantitative analysis of colony formation assay showing the number of colonies in the control and NR4A3-overexpression cells.

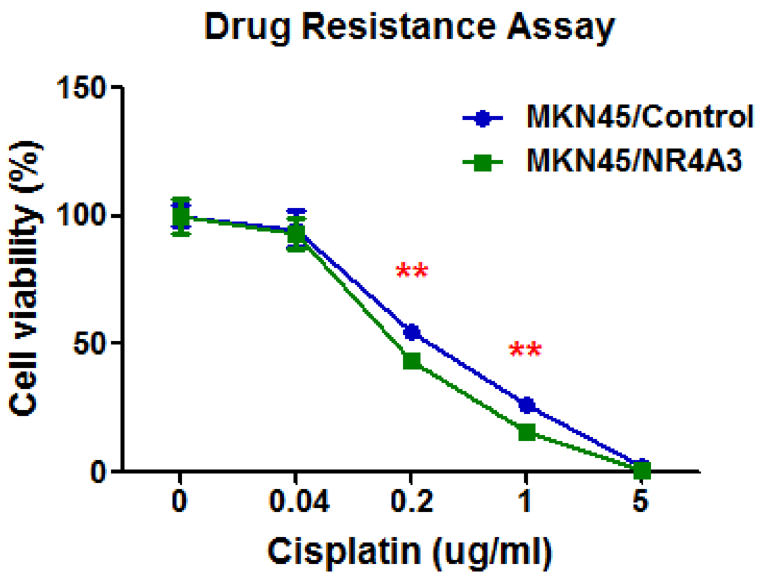

Figure S5. Ectopic expression of *NR4A3* sensitized the cells to chemotherapeutic agent. MKN45 gastric cancer cells transfected with empty or NR4A3 expression vector were treated with vaious concentration of cisplatin for 72 hr. Cell viability was determined by CCK8 assay.

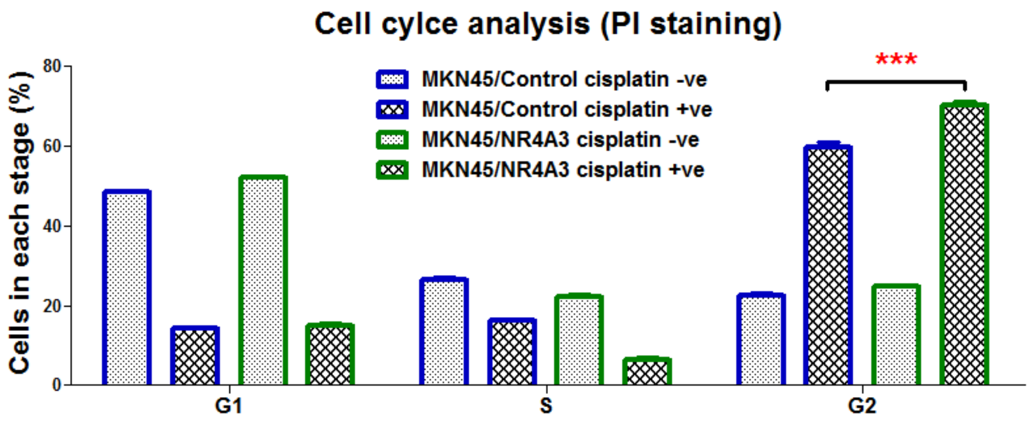

Figure S6. Ectopic expression of *NR4A3* enhanced G2/M arrest in MKN45 cells treated with cisplatin. MKN45 cells transfected with empty or NR4A3 expression vector were treated with 1μg/ml of cisplatin for 24 hr. Population of cells at each phase of the cell cycle were analyzed by FACScan flow cytometer. ***, P<0.005
